# A new genus and species of leaf-mining moth from the French Alps, *Mercantouria neli* gen. n., sp. n. (Lepidoptera, Gracillariidae)

**DOI:** 10.3897/zookeys.586.8375

**Published:** 2016-05-04

**Authors:** Peter Huemer, Carlos Lopez-Vaamonde, Paolo Triberti

**Affiliations:** 1 Tiroler Landesmuseen Betriebsgesellschaft m.b.H., Naturwissenschaftliche Sammlungen, Feldstr. 11 a, A-6020 Innsbruck, Austria; 2 INRA, UR0633 Zoologie Forestière, F-45075 Orléans, France; 3 Institut de Recherche sur la Biologie de l’Insecte, CNRS UMR 7261, Université François-Rabelais de Tours, UFR Sciences et Techniques, 37200 Tours, France; 4 Museo Civico di Storia Naturale, Lungadige Porta Vittoria 9, I-37129 Verona, Italy

**Keywords:** COI, DNA barcoding, histone 3, Gracillaria group, new genus, new species, Alps

## Abstract

The Alps are a hotspot of biodiversity in Europe with many Lepidoptera species still to be discovered. Here we describe a new gracillariid genus and species, *Mercantouria neli* **gen. n.** and **sp. n.** The morphology of the male genitalia is highly differentiated with unique features. DNA barcodes show that its nearest neighbor is the North American species ‘Caloptilia’ scutellariella (Braun, 1923). *Mercantouria neli* is known from four adults (two males and two females) collected at two localities in the French Alps. Its host plant and life cycle remain unknown.

## Introduction

For more than two centuries the Alpine Lepidoptera fauna has been at the focus of intense taxonomic and faunistic work. As a result, an estimated 5000 Lepidoptera species are known to occur in the Alps, of which about 250 species (ca. 5%) but only a single monotypic genus (*Lunakia* Klimesch, 1941, Plutellidae) are known to be endemic to the alpine region (Huemer 1998, unpubl. data). Several additional genera from various families, e.g. *Kessleria* Nowicki, 1864 (Yponomeutidae), *Sattleria* Povolný, 1965 (Gelechiidae), *Sphaleroptera* Guenée, 1845 (Tortricidae), *Erebia* Dalman, 1816 (Nymphalidae), *Sciadia* Hübner, 1822 and *Glacies* Milliére, 1874 (Geometridae), show strong diversification and endemism in the alpine region. However, despite the relatively good knowledge of the Alpine Lepidoptera fauna, the recent use of DNA barcoding has helped to reveal an increasing number of new species. Many of these newly discovered taxa are cryptic or morphologically difficult to distinguish (Buchner 2015; Huemer 2011; Huemer and Hausmann 2011; Huemer and Hebert 2011; Huemer et al. 2013; Huemer and Timossi 2014; Huemer et al. 2014a,b; Huemer and Mutanen 2015; Kirichenko et al. 2015; Tabell and Baldizzone 2014; Whitebread 2007; Zeller and Huemer 2015). However, here we report the remarkable discovery of a genetically and morphologically highly divergent micro moth species of the family Gracillariidae from the French Alps.

Gracillariidae are a relatively well known family in Europe with 23 genera and 260 species recorded (De Prins and De Prins 2015). However, new species have been discovered recently (Laštůvka and Laštůvka 2006; 2012; Triberti 2007; Laštůvka et al. 2013; Kirichenko et al. 2015).

The new genus and species described here belongs to the Gracillariinae. This subfamily contains four groups of genera: *Acrocercops*, *Gracillaria*, *Parectopa* and *Parornix* (Kumata et al. 1988a,b). The new taxon belongs to the *Gracillaria* group, which is characterized by the presence of the vein R_2+3_ on the hindwing (Kumata 1982). In the Western Palearctic eight genera are recognized to belong to the *Gracillaria* group: *Gracillaria* Haworth, 1828; *Caloptilia* Hübner, 1825; *Povolnya* Kuznetzov, 1979; *Calybites* Hübner, 1822; *Euspilapteryx* Stephens, 1835; *Aspilapteryx* Spuler, 1910; *Aristaea* Meyrick, 1907; *Cupedia* Klimesch & Kumata, 1973 (Kumata 1982; 1995). In this study also the monotypic Eastern Palearctic genus *Eucalybites* Kumata, 1982, has been included in the comparison for some similarities.

To date, over 40 species of the *Gracillaria* group are known to occur in Europe, about 30 included in the genus *Caloptilia*. In the larval stage most species are leaf miners in early instars and leaf rollers in late instars, while some are leaf miners throughout the whole feeding stage. The majority of species prefer the leaves of bushy and woody plants, included mainly in the families Aceraceae and Betulaceae (especially favored), Fagaceae, Oleaceae and Anacardiaceae. More rarely they also feed on herbaceous plants, particularly in the families Plantaginaceae, Hypericaceae and Asteraceae (De Prins and De Prins 2015).

Here we present genetic (mitochondrial and nuclear) and morphological data that support the hypothesis that individuals of a highly differentiated *Gracillariinae* collected in the French Alps represent a distinct lineage that we formally describe as a new genus and a new species – *Mercantouria neli* Huemer, Lopez-Vaamonde & Triberti, gen. n., sp. n.

## Materials and methods

### Taxon sampling

Specimens examined in this study were obtained by light trapping integrating UV tubes and mercury lamp. A single specimen was collected flying freely above low vegetation at dusk. Specimens were preserved in tubes, pinned and wings spread in the next morning.

### Morphology and nomenclature

We examined the morphology of four dried, pinned specimens belonging to *Mercantouria neli*. The holotype was photographed with an Olympus SZX 10 binocular microscope and an Olympus E 3 digital camera and processed using the software Helicon Focus 4.3 and Adobe Photoshop CS4 and Lightroom 2.3. Genitalia photographs were taken with an Olympus E1 Digital Camera from Olympus BH2 microscope.

Genitalia dissections and slide mounts followed Robinson (1976). Terminology of the genitalia follows Klots (1970) and Kristensen (2003); wing venation Kumata (1982).

Type material is deposited in the collection of TLMF = Tiroler Landesmuseum Ferdinandeum, Innsbruck, Austria.

### DNA sequencing and analysis

DNA extracts were prepared from a single hind leg removed from three of the four specimens of *Caloptilia neli*. DNA extraction, PCR amplification and sequencing of the barcode region were carried out at the Canadian Centre for DNA Barcoding (CCDB, Biodiversity Institute of Ontario, University of Guelph) following standard protocols (deWaard et al. 2008). Sequence divergences were quantified using the Kimura 2-parameter model implemented within the analytical tools on BOLD (www.boldsystems.org) (Ratnasingham and Hebert 2007).

In addition, an aliquot of DNA of sample TLMF Lep 08375 was received from CCDB (Guelph). Because DNA concentration was low (0.28 ng/μl), we performed a whole genome amplification using REPLI-g Mini Kit (Qiagen). Then a 350 bp fragment of the nuclear gene histone H3 was sequenced using primers and PCR conditions as described in Kirichenko et al. (2015). This was done at Marko Mutanen’s lab (University of Oulu, Finland).

To explore the phylogenetic position of the new species and its generic classification we combined the mitochondrial and nuclear data for *Mercantouria neli* with a published dataset of 39 Gracillariidae species and one outgroup (Kirichenko et al 2016, Gutzwiller et al. 2015; Kawahara et al. 2011) (Suppl. material 1). All new specimens and sequence data are available in BOLD in the public dataset dx.doi.org/10.5883/DS-CAYOLLE. Sequences are also deposited in GenBank and accession codes are provided in Suppl. material 1. Sequences were concatenated and aligned using Geneious 9.05 (http://www.geneious.com/).


 Maximum parsimony (MP) and maximum likelihood (ML) analyses were performed using PAUP* version 4.0 a 147 (Swofford 2002).

## Results

### Morphology

#### Mercantouria

Taxon classificationAnimaliaLepidopteraGracillariidae

Huemer, Lopez-Vaamonde & Triberti gen. n.

http://zoobank.org/4B34364D-EDD2-4E73-A2A8-903EC332015C

**Figure 1.**
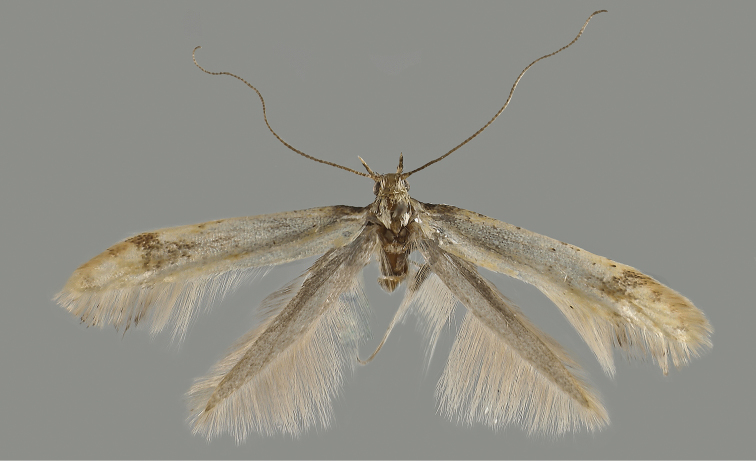
*Mercantouria neli* sp. n., holotype; France, Dep. Alpes-Maritimes, Col de la Cayolle N, 2080 m, 19.7.2013, leg. Mayr.

**Figure 2.**
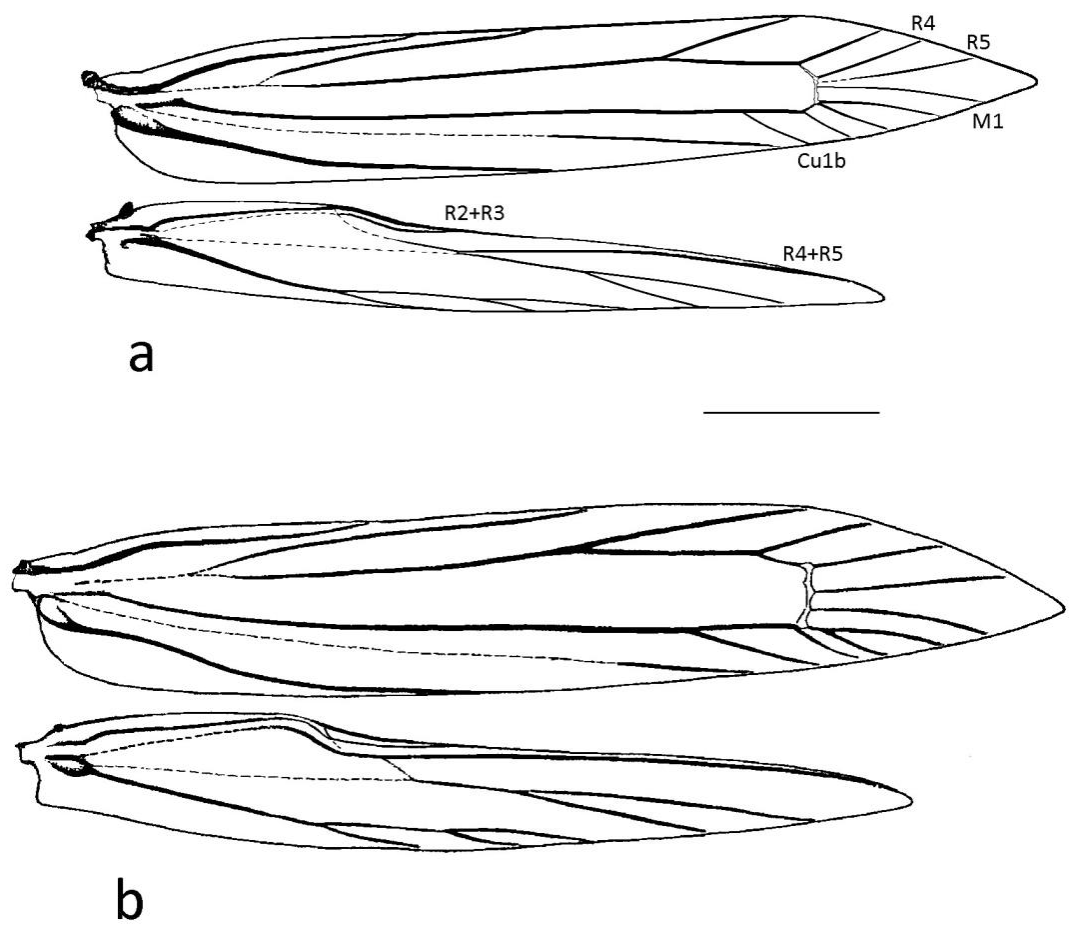
Wing venation: **a** *Mercantouria neli* sp. n.; **b** *Caloptilia stigmatella* (Fabricius, 1781). Scale length = 1 mm.

**Figure 3.**
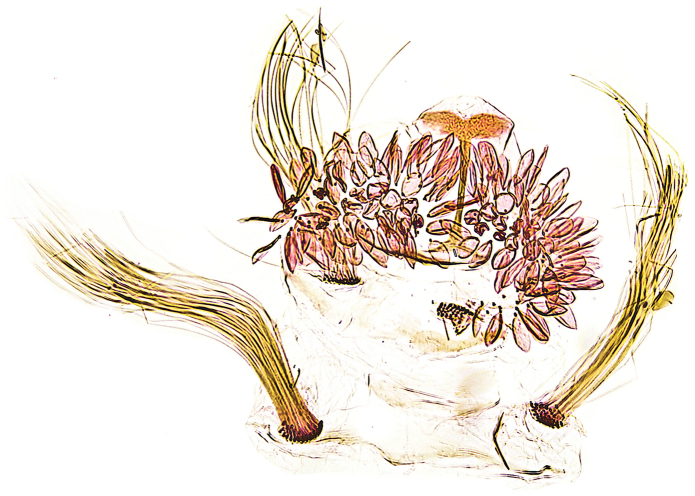
*Mercantouria neli* sp. n., holotype, abdominal segments 7–8; France, Dep. Alpes-Maritimes, Col de la Cayolle N, 2080 m, 19.7.2013, leg. Mayr; genitalia slide P.Huemer TIN 94 ♂.

**Figures 4–5.**
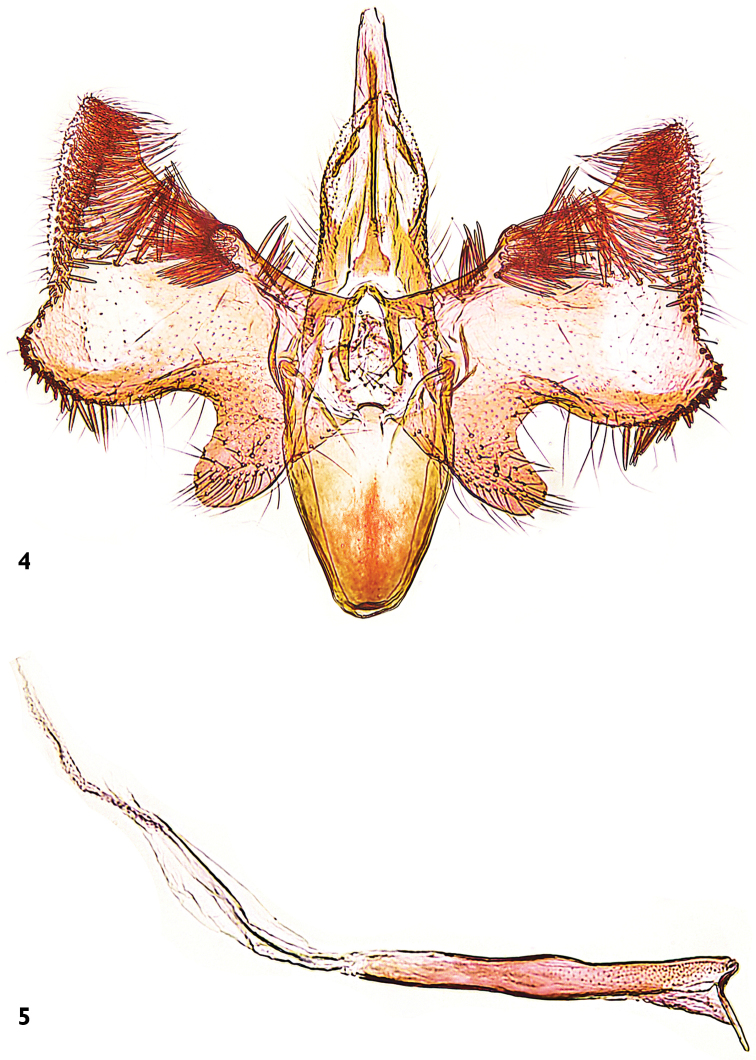
*Mercantouria neli* sp. n., holotype, male genitalia; France, Dep. Alpes-Maritimes, Col de la Cayolle N, 2080 m, 19.7.2013, leg. Mayr; genitalia slide P.Huemer TIN 94 ♂ **4** tegumen-vinculum-valva complex **5** phallus.

**Figures 6–7.**
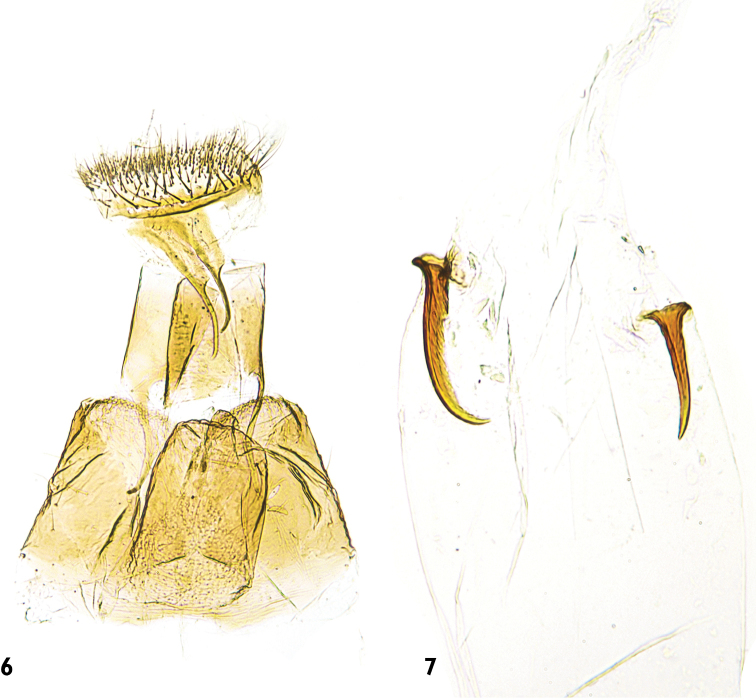
*Mercantouria neli* sp. n., paratype, female genitalia; France, Alpes-Maritimes, N Col de la Cayolle, Col de la Boucharde N, 1950m, 7.7.2012, leg. Huemer; genitalia slide P.Huemer TIN 93 ♀ **6** last segments **7** corpus bursae-signa.

Figs 1 , 2 , 3 , 4–5 , 6–7

##### Type species.

*Mercantouria neli* Huemer, Lopez-Vaamonde & Triberti, sp. n.

##### Description.

**Adult** (Fig. 1). Forewing length 5.1–5.8 mm. Head. Vertex and face loosely scaled; ocelli absent; proboscis naked, well developed. Antenna about as long as forewing, smooth, each flagellomere with an annulus of slender scales basally and another of shorter scales at apex, about 0.2× length of basal ones, completely covered by the first (Triberti 1998); scape moderate, about 3.0× length of pedicel, pecten missing. Labial palpus long, upturned, pointed apically, segment 2 as long as apical one, slightly thickened with scales towards apex. Maxillary palpus smooth, shorter than apical segment of labial palpus.

Thorax. Smoothly scaled. Forewing narrow, lanceolate; discoidal cell with distal margin nearly vertical, 13-veined; all radial veins separated but vein R4, R5 and M1 very close at their bases; veins M2 and M3 connate and arising from lower angle of cell; Cu1b arising from cell more apical than R2. Hindwing narrowly lanceolate, 8-veined; cell opened between M2 and M3, R4+R5 not parallel to the costal margin and meeting directly with M1+M2 (Fig. 2). Legs with tibial spur pattern 0-2-4; epiphysis present but partly hidden by long scales.

Abdomen. In the male segment 7 and 8 weakly membraneous, with a pair of coremata on each segment; anterior pair of coremata consisting of hairlike scales, longer and thicker than the posterior pair (Fig. 3). Sternum and tergum 7 reduced into a thin sclerites; sternum 8 also reduced but tergum is formed by a small, fan-shaped sclerite, with a narrow median ridge. Female postabdominal segments unmodified.

Male genitalia (Figs 4–5). Tuba analis produced beyond tegumen, membraneous, with a narrowly sclerotized subscaphium, widened basally. Tegumen weakly sclerotized, simple. Valva stout, with sacculus distinctly protruded and rounded apically, setose; cucullus straight, upturned, covered with strong setae on dorso-distal area; costal margin irregular with similar setae medio-distally. Diaphragma with some fine setae at base of anellus. Phallus slightly shorter than valva, apically with long rod-like sclerite branching off at right angle, no cornuti are visible.

Female genitalia (Figs 6–7). Lamella postvaginalis not connected with apophyses anteriores. Ostium bursae located under a lobate sternite 7. Ductus bursae completely membraneous, slender; corpus bursae ellipsoidal with two curved sickle-shaped signa, one of which is slightly longer than the other.

##### Etymology.

The generic name refers to the region of Mercantour National Park (France).

##### Diagnosis.

*Mercantouria* belongs to the *Gracillaria* group for the presence of a very short vein R_2+3_ in the hindwing, running in parallel with apical part of vein Sc+R_1_. Moreover this new genus shares with most genera of the *Gracillaria* group the following characters: legs more or less smooth-scaled except for mid femur and tibia thickened with raised scales; forewing 13-veined with M_2_ and M_3_ connate, R_1_ arising from cell near base of wing, upper vein of cell weakened on basal part just beyond the point where R_1_ branches off; hindwing 8-veined, with radial veins always 2-branched, veins M_1_ and Cu_1a_ stalked with veins M_2_ and Cu_1b_ respectively, vein M_3_ branched from vein Cu_1a_, cell opened between M_2_ and M_3_; in male genitalia abdominal segment 7 and 8 weakly membraneous, each of them having a pair of coremata which are in a bundle of long and hairy scales, the latter covered with more or less deformed scales; in female, corpus bursae with two large sickle-shaped signa.

Within *Gracillaria* group the genera are difficult to identify on the basis of apomorphies and comparisons are rather complicated due to the “cross” distribution of characters. *Mercantouria* shows some similarity to the genus *Caloptilia* and allied genera (*Gracillaria*, *Povolnya*, *Euspilapteryx*, *Aspilapteryx* and *Eucalybites*): (1) forewing 13-veined and hindwing 8-veined, albeit with slight differences in the relative positions of some veins; also in *Gracillaria* and *Povolnya* there is a similar venation but in the former there are strong differences in the pregenital segments, the segment 7 being like the preceding and without coremata and the latter with peculiar male genitalia, with the tegumen having a pair of peniculi projected from caudal margin of tegumen; (2) male abdomen with two pairs of coremata more or less similar in length and thickness; a similar condition is found in *Povolnya* and *Euspilapteryx* but the latter differs from the new species in the forewing venation (12-veined) and female genitalia (only one signum); (3) in the female genitalia, the bursa copulatrix has two corniform signa; this character is shared with *Aspilapteryx* and *Eucalybites* however both differ from forewing venation (12-veined) and coremata of different size or only one pair.

**Figure 8.**
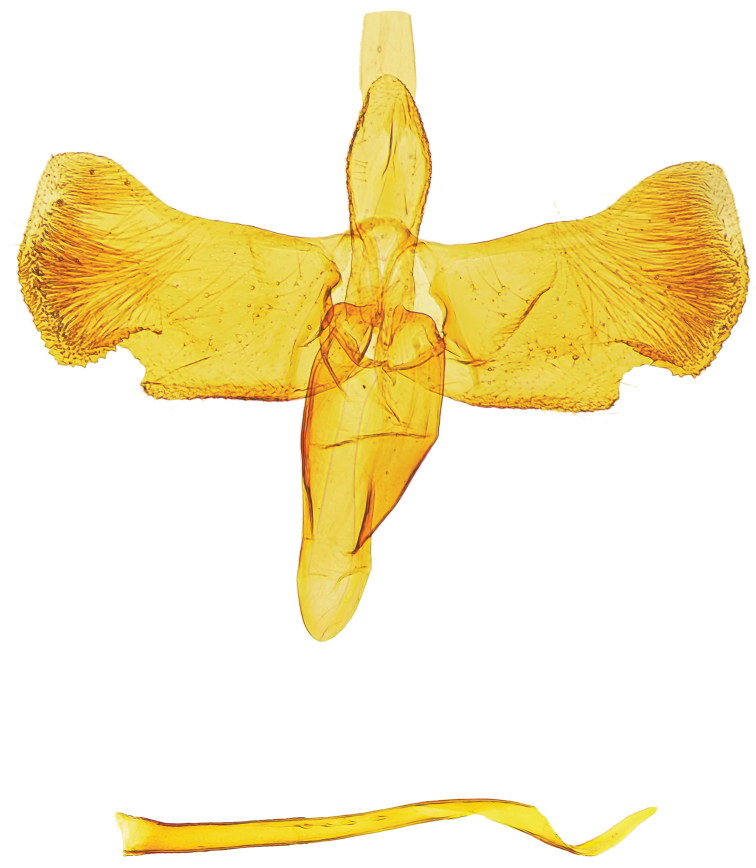
*Aspilapterix spectabilis*, paratype, male genitalia; Austria, Osttirol, Virgental, Venedigergruppe, Sajatmähder, 2150–2350 m, 31.7.1993, leg. Ryrholm; genitalia slide P.Huemer TIN 33 ♂.

*Mercantouria* differs from these genera in the following morphological characters: 1) the forewings show veins R_4_, R_5_ and M_1_ very close, weakened or obsolescent at their bases; this character is unknown within *Gracillaria* group and it seems closer to *Acrocercops* group. 2) The hindwings show veins R_4_+R_5_ directly connected with radial vein and divergent from costa, this condition is only similar to *Eucalybites* and *Aspilapteryx*; however both clearly differ by having the forewing 12-veined. 3) Lack of pecten which also occurs in a few taxa closely related to *Caloptilia*: subgenera *Timodora* Meyrick, 1886, *Phylloptilia* Kumata, 1982 and the genus *Povolnya*, sometimes considered as another subgenus of *Caloptilia* (Kumata 1982). 4) The male genitalia has a highly modified valva (Fig. 4) unlike any other known in the genus *Caloptilia*; only a somewhat similar shape of the valva is seen in *Aspilapteryx spectabilis* (Fig. 8) (Huemer 1994) and *Eucalybites aureola* (Kumata 1982) but easily distinguishable from the cucullus, which is straight and covered with strong setae along its margin in the new genus.

#### Mercantouria neli

Taxon classificationAnimaliaLepidopteraGracillariidae

Huemer, Lopez-Vaamonde & Triberti sp. n.

http://zoobank.org/FF3D1062-2586-4C50-AE95-440A1AC90230

Figs 1 , 2 , 3 , 4–5 , 6–7

##### Type material.

Holotype ♂ (Fig. 1): “Frankreich Dep. Alpes-Maritimes Col de la Cayolle N 6°44'21"E, 44°16'49"N 2080 m, 19.7.2013 leg. Mayr” “P.Huemer TIN 94 ♂” “DNA Barcode TLMF Lep 16937” (TLMF).

Paratypes: 1 ♀, Frankreich, Alpes-Maritimes, N Col de la Cayolle, Col de la Boucharde N, 6°44'36"E, 44°17'0"N, 1950m, 7.7.2012 leg. Huemer, TLMF 2013-010 (gen.slide P.Huemer TIN 93 ♀; DNA Barcode ID TLMF Lep 08375); 1 ♂, Frankreich, Alpe-Maritimes, PN Mercantour, 2115 m, Col de la Cayolle Nord, N44°16,78', E6°44,32', 21.7.2014, leg. Drouet (gen. slide P.Huemer TIN 95 ♂; DNA Barcode ID TLMF Lep 16938); 1 ♀, Htes-Alpes, Ristolas, La Roche Ecroute, 1750 m, 12.7.2010, leg. Nel, genitalia slide 24139 J. Nel (all coll. TLMF).

##### Description

(Fig. 1). Head. Labial palpus pale ochre-yellowish, apical segment dark brown medio-basally. Legs smooth scaled, dark brown with exception of hind leg that are lighter; all tarsi white.

Thorax. Dorsum and tegulae ochre yellow. Forewing pale ochre yellow with small spots or suffusion of dark brown, mostly along the discoidal cell and sometimes forming, in the apical third of the wing, an irregular fascia. Hindwing light ochre-greyish.

Abdomen, male and female genitalia. See under the genus description.

##### Etymology.

Named in honour of Dr. Jacques Nel (La Ciotat, France) who independently recognized and collected the new species.

##### Diagnosis.

Superficially the adult of *Mercantouria neli* can be confused with some members of the *Gracillaria* group, like light coloured specimens of *Caloptilia roscipennella* (Hübner, 1796) and *Aspilapteryx limosella* (Duponchel, 1843). However, in both species a trace remains of neat rows of darkish small spots, along the costa in the former and in the middle of wing in the latter, while in *Mercantouria neli* the dark scales create confused and ill-defined spots. In the male genitalia, the short valva with a protruded sacculus shows some affinity to *Aspilapteryx* and *Eucalybites* species, particularly *Aspilapteryx spectabilis* and *Eucalybites aureola*. However, the new species can be easily separated by the straight cucullus and the numerous, thickened setae along its margin and costa. The female genitalia are easily distinguishable from other species of the *Gracillaria* group by the heavily sclerotized sternum 7, which is flap shaped, lobate on caudal margin and about as long as tergum 7. A similar structure is present in *Eucalybites aureola* but with the sternum 7 much narrower, about half of tergum, and a heavily sclerotized sterigma with a complicated shape (Kumata 1982).

##### Molecular data.

We obtained DNA barcode data for all 39 individuals and H3 data for 32 out of the 39 samples (Suppl. material 1). The three DNA barcodes obtained for *Mercantouria neli* (maximum intraspecific distance = 0.49%) fall within the same Barcode Index Number (BOLD:ACA9784) allowing the unequivocal identification of the new species. The nearest neighbor is the North American species *Caloptilia scutellariella* (Braun, 1923) (BOLD:AAU2901) and associated or possibly misidentified DNA clusters (BOLD:ABX8283, BOLD:AAP8031) at a genetic distance of 8.41%.That would suggest that the new species could be a representative of the genus *Caloptilia*. However, the generic assignment of *Caloptilia scutellariella* seems doubtful from genitalia morphology and needs further revision.

**Figure 9.**
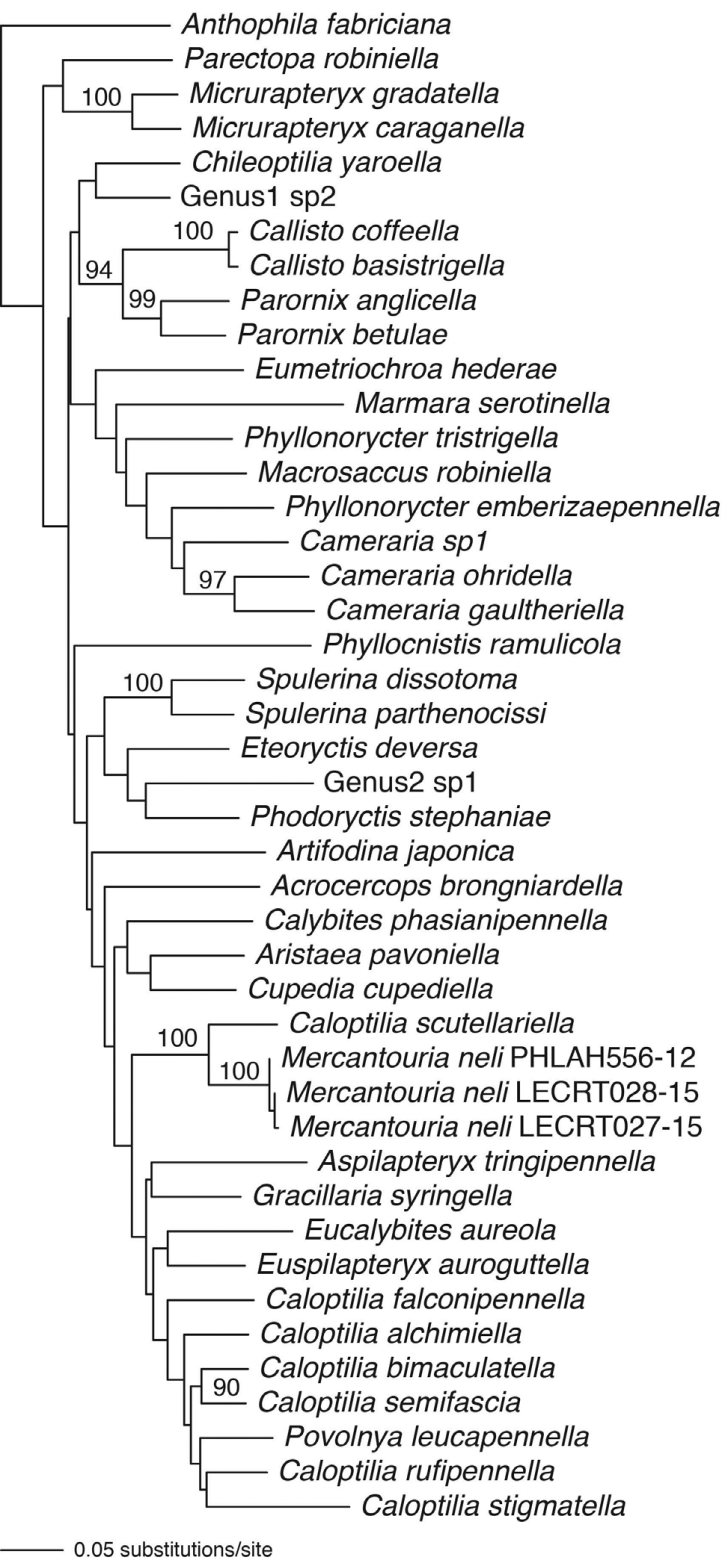
Maximum likelihood tree based on COI and H3 sequences for 43 Gracillariid species. –ln likelihood = 11983.13. Bootstrap values are indicated for nodes more than 50% support (1000 replications). The general time reversible model of sequence evolution was used with the following settings: LSet nst=6 rclass=(abcdef) rmatrix=(2.9833028 10.54651 15.145156 9.0102019 24.833933) basefreq=(0.26825699 0.20834936 0.17265202) rates=gamma shape=1.0542648 pinv=0.53477118).

**Figure 10.**
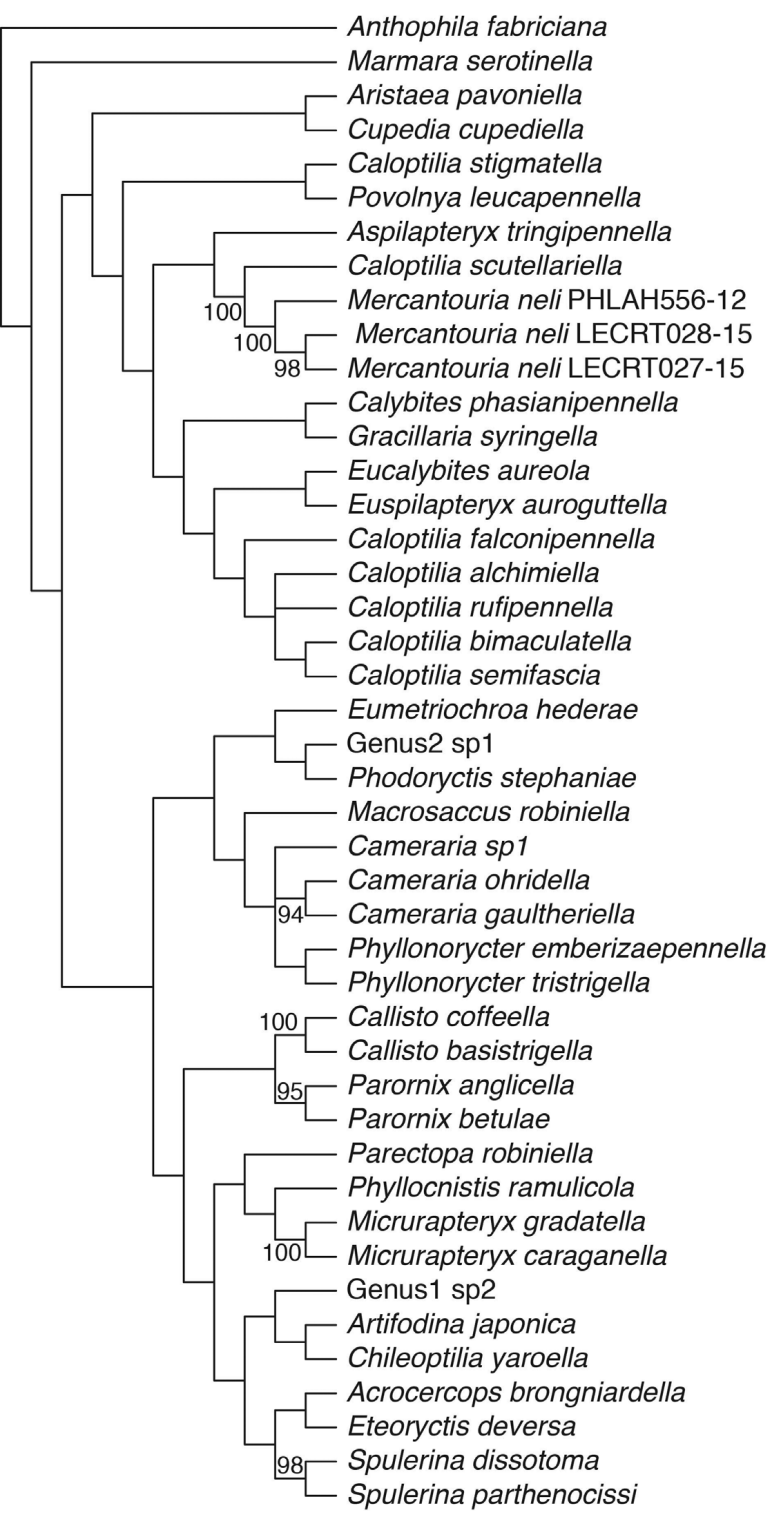
Semistrict consensus tree of four most parsimonious trees (length 2618, consistency index (CI) 0.272, and retention index (RI) 0.385). Bootstrap values are indicated for nodes more than 50% support (1000 replications).

The ML analysis shows that the new species falls within a clade formed by six *Caloptilia* species and *Gracillaria syringella*, although with low bootstrap support (Fig. 9). MP analysis returned four most parsimonious trees. The semistrict consensus is shown in Fig. 10.

##### Biology.

**Figure 11.**
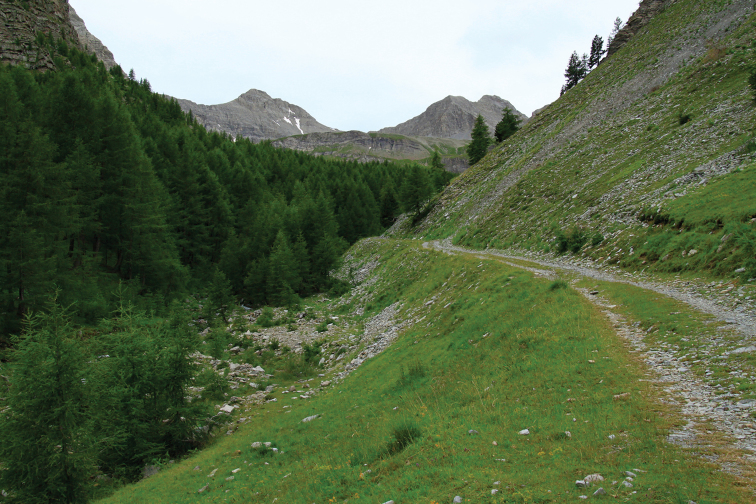
Type locality of *Mercantouria neli* sp. n. near Col de la Cayolle.

Host-plant and early stages are unknown. *Mercantouria neli* was collected only in singletons so far, either at dusk or during the night at light. The flight period seems to be short, lasting from mid- to late July. The habitat (Fig. 11) is dominated by subalpine scree and grassland on limestone soil. Vertical distribution: from about 1750 to 2100 m.s.l.

##### Distribution.

The new species is so far known from a small area of the French Hautes-Alpes and Alpes-Maritimes.

## Discussion

The description of a new genus is an arbitrary decision (Hennig 1966; Humphreys and Barraclough 2014) and a particularly difficult one to make when the genus is monotypic. We based our decision on the fact that neither morphological nor DNA sequence data support the placement of the new taxon within any of the extant Gracillariidae genera. Indeed, the highly differentiated male genitalia with unique structures of the valva, and the forewing venation support the hypothesis of a new genus. Both mitochondrial and nuclear sequence data show that the new taxon might be closely related to the genus *Caloptilia* and in particular to the North American *Caloptilia scutellariella*. However, we think that *Caloptilia scutellariella* belongs most likely to a different genus, not *Caloptilia*, but more data is needed to test this hypothesis.

*Mercantouria neli* could represent a non native species introducted into the Alps. Indeed there are several species of non-native Gracillariidae established in Europe (Lopez-Vaamonde et al. 2010). However, based on the repeated collection of several individuals in different years in such remote alpine habitat we think an anthropogenic introduction is highly unlikely.

*Mercantouria neli* most likely represents a xero-montane relict alpine species like the recently discovered *Callisto basistrigella* (Kirichenko et al. 2015). However, gracillariid species thought to be endemic to the Alps such as *Aspilapteryx spectabilis* have been discovered in other mountain ranges (Huemer 2011) and thus further work is needed to confirm the endemism status of *Mercantouria neli* in the Alps.

Like other alpine Lepidoptera such as the recently described *Syrianarpia faunieralis* Gianti, 2005 (Crambidae), a species endemic to the Cottian Alps but with congeneric relatives in Turkey and on the Krim peninsula (Gianti 2005; Goater et al. 2005; Huemer 2009), *Mercantouria neli* could also have its closest relatives in Asia. Indeed, there is an undescribed species of a Gracillariinae collected in Turkey (specimens deposited at the Natural History Museum in Copenhagen) whose morphology shows some affinities to *Mercantouria neli* (unpublished morphological data). However, genetic data is necessary to support potential congenerity of these two taxa.

Finally, additional biological and molecular data are needed to understand the interrelationships of *Mercantouria neli* with the other genera within the *Gracillaria* group.

## Supplementary Material

XML Treatment for Mercantouria

XML Treatment for Mercantouria neli

## Supplementary materials

Supplementary material 1 Sample information for specimens used in this study Data type: Species data Explanation note: Details of collecting data, images, sequences, and trace files for the barcoded specimens are available in the public BOLD dataset “DS-CAYOLLE”, accessed at http://dx.doi.org/10.5883/DS-CAYOLLE This dataset is made available under the Open Database License (http://opendatacommons.org/licenses/odbl/1.0/). The Open Database License (ODbL) is a license agreement intended to allow users to freely share, modify, and use this Dataset while maintaining this same freedom for others, provided that the original source and author(s) are credited.Peter Huemer, Carlos Lopez-Vaamonde, Paolo Triberti
